# Clinical-neuroimaging-pathological relationship analysis of adult onset Neuronal Intranuclear Inclusion Disease (NIID)

**DOI:** 10.1186/s12883-022-03025-1

**Published:** 2022-12-15

**Authors:** Chenhui Mao, Liangrui Zhou, Jie Li, Junyi Pang, Shanshan Chu, Wei Jin, Xinying Huang, Jie Wang, Caiyan Liu, Qing Liu, Honglin Hao, Yan Zhou, Bo Hou, Feng Feng, Lu Shen, Beisha Tang, Bin Peng, Liying Cui, Jing Gao

**Affiliations:** 1 Department of Neurology, State Key Laboratory of Complex Severe and Rare Diseases, Peking Union Medical College Hospital, Chinese Academy of Medical Science/Peking Union Medical College, Shuaifuyuan 1St, Dongcheng District, Beijing, 100730 China; 2 Department of Pathology, Peking Union Medical College Hospital, Chinese Academy of Medical Science/Peking Union Medical College, Beijing, 100730 China; 3 Department of Radiology, Peking Union Medical College Hospital, Chinese Academy of Medical Science/Peking Union Medical College, Beijing, 100730 China; 4 Department of Neurology, National Clinical Research Center for Geriatric Disorders, Xiangya Hospital, Central South University, Changsha, 410008 Hunan China

**Keywords:** Neuronal intranuclear inclusion disease, Diffusion weighted imaging, Leukoencephalopathy, Dementia, Kite-like

## Abstract

**Background:**

Neuronal Intranuclear Inclusion Disease (NIID) is a degenerative disease with heterogeneous clinical manifestations. We aim to analysis the relationship between clinical manifestations, neuroimaging and skin pathology in a Chinese NIID cohort.

**Methods:**

Patients were recruited from a Chinese cohort. Detail clinical information were collected. Visual rating scale was used for evaluation of neuroimaging. The relationship between clinical presentations and neuroimaging, as well as skin pathology was statistically analyzed.

**Results:**

Thirty-two patients were recruited. The average onset age was 54.3 y/o. 28.1% had positive family history. Dementia, autonomic nervous system dysfunction, episodic attacks were three main presentations. CSF analysis including Aβ_42_ and tau level was almost normal. The most frequently involved on MRI was periventricular white matter (100%), frontal subcortical and deep white matter (96.6%), corpus callosum (93.1%) and external capsule (72.4%). Corticomedullary junction DWI high intensity was found in 87.1% patients. Frontal and external capsule DWI high intensity connected to form a “kite-like” specific image. Severity of dementia was significantly related to leukoencephalopathy (*r* = 0.465, *p* = 0.0254), but not cortical atrophy and ventricular enlargement. Grey matter lesions were significantly associated with encephalopathy like attacks (*p* = 0.00077) but not stroke like attacks. The density of intranuclear inclusions in skin biopsy was not associated with disease duration, severity of leukoencephalopathy and dementia.

**Conclusions:**

Specific distribution of leukoencephalopathy and DWI high intensity were indicative. Leukoencephalopathy and subcortical mechanism were critical in pathogenesis of NIID. Irrelevant of inclusion density and clinical map suggested the direct pathogenic factor need further investigation.

## Background

Neuronal Intranuclear Inclusion Disease (NIID) is a systemic degenerative disease characterized by the presence of eosinophilic intranuclear inclusions in neurons, glial cells and systemic visceral cells [1]. The clinical phenotype of NIID is heterogeneous. It can be classified into sporadic and familial types based on familial inheritance, or into infantile-onset, juvenile onset, and adult-onset types based on age of onset [2]. The classical presentation of sporadic adult onset NIID is progressive dementia and widespread leukoencephalopathy, however, episodic encephalopathy or stroke like attacks, parkinsonism or tremor, autonomic nervous system dysfunction, headache, neuromuscular disorders are all reported [1, 2].

In the flowchart proposed by Sone et al., corticomedullary junction diffusion weighted imaging (DWI) high intensity and ubiquitin positive intranuclear inclusions in adipocytes, fibroblasts and sweat gland cells via skin biopsy were specific features of NIID [3]. In 2019, several groups reported GGC repeat expansion in the 5′ region of human specific *NOTCH2NLC* gene was the genetic mechanism of NIID, which promoted the recognize and early diagnosis of the disease [4–7].However, the pathogenesis of the disease as well as the component of the inclusions are not clear, which interferes with the understanding of the complicated clinical and neuroimaging manifestations. Here we aim to analysis the relationship between clinical manifestations, neuroimaging and skin pathology based on systemically describing the clinical and neuroimaging presentation of a Chinese NIID cohort. The findings of our study might help better understanding and further research of NIID in the near future.

## Methods

### Patients inclusion

All patients were recruited from Dementia and Leukoencephalopathy Clinic, Department of Neurology, Peking Union Medical College Hospital, between Jan 2017 and Dec 2021. The diagnosis of NIID was made based on clinical presentation, neuroimaging and skin pathology with/out *NOTCH2NLC* gene expansion as suggested by Sone et al. [3]. Clinical histories including demographic information, onset age, duration of disease, symptoms, family history were taken. Necessary laboratory tests including thyroid function, liver and kidney function, homocysteine, α-galactosidase, β-galactosidase, galactocerebrosides, arylsulfatase and *FMR1* gene expansion were carried out for differential diagnosis. Mini Mental State Examination (MMSE), Montreal Cognitive Assessment-PUMCH edition (MoCA-PUMCH edition) [8], Activities of Daily Living (ADLs) were evaluated for cognitive function of the patients. Cerebrospinal fluid examination, electroencephalogram, Electromyogram, ^18^F-FDG-PET/CT were done in part of the patients. Commercial accessible ELISA kits were utilized for measurement of CSF t-tau, p-tau, Amyloid β 42 (Aβ42) respectively by INNOTEST Tau ELISA, Phospho-tau, and Aβ42 (Fujirebio, Ghent, Belgium).

### Ethics and consents

The study was conducted according to declaration of Helsinki in 1964 and the subsequent revisions. The study was approved by the Ethics Committee of Peking Union Medical College Hospital (No. JS1836). All patients and caregivers provided written informed consent.

### Neuroimaging evaluation

Brain magnetic resonance imaging (MRI) examinations were performed using a 3 T MRI scanner (Avanto, Siemens, Erlangen, Germany). Axial T1-weighted, T2-weighted, Fluid attenuated inversion recovery (FLAIR) and diffusion weighted imaging (DWI) were performed. In part of the patients, contrasted imaging with gadopentetate dimeglumine and follow up imaging were collected. Visual rating scale was used for evaluation of cortical atrophy, ventricular enlargement and white matter hyperintensities. Cortical atrophy was evaluated with general cortical atrophy (GCA) scale [9, 10]. Score 0,1,2,3 meant no atrophy, widen sulcus, atrophied gyrus and knife edge gyrus, respectively. Ventricular enlargement was evaluated with scale defined by O’Donavan et al. [11], grading into 0,1,2,3 based on the height, width and size of lateral horn. White matter hyperintensities were evaluated with scale defined by Scheltens et al. [12]. White matter lesions were divided into four areas, including periventricular white matter, deep white matter, basal ganglia and infra-tentorial white matter. Scores were given based on size and count of lesions, then summarized.

### Skin pathology

Skin biopsies were performed after informed consent. After local anesthesia with lidocaine, a 5 mm diameter biopsy specimen was obtained at 10 cm above the lateral malleolus. All samples were fixed in 10% formalin and then embedded in paraffin, sectioned into 6 mm thickness [3]. All sections were stained with haematoxylin & eosin (H&E). Immunohistochemical stain was performed with anti-ubiquitin (1B4-UB, Abcam, UK) and anti-p62 (EPR18351, Abcam, UK) antibodies. Images were acquired by upright digital microscope (BX43, Olympus, Japan) and scale Bar was edited with ImageJ. Quantitative analysis was to calculate the largest number of immunohistochemical positive inclusion bodies in a 400 × magnified visual field.

### NOTCH2NLC gene analysis

Venous blood was taken into vacuum tubes with Ethylene diamine tetra acetic (EDTA) coagulant. Long read genome sequencing and GGC repeat size determination was conducted at National Clinical Research Center for Geriatric Disorders, Xiangya Hospital, Central South University, China. The protocol and methods were described previously by Tian et al. [5]. GGC repeat size larger than 66 was positive for NIID.

### Statistical analysis

Statistical analyses were performed using SPSS V.25.0 software. Measurement data were described as average ± standard deviation, and numeration data were described as ratio. As several groups of data were not normal distribution, nonparametric analysis was conducted. Measurement data were statistically analyzed with the Mann–Whitney test (2 groups) and Kruskal Wallis (3 groups), while numeration data were analyzed with the χ^2^ test. Statistical graphs were produced with GraphPad Prism. The level of statistical significance was set to *p* < 0.05. Spearman’s correlation coefficient and linear regression were performed to compare the relationship between dementia and white matter lesions.

## Results

### Demographic and clinical information

Thirty-two patients were recruited. 20 patients finished *NOTCH2NLC analysis and* 19 patients had expanded *NOTCH2NLC* GGC repeat. Positive pathological and negative gene result was found in the 1 patient. Twenty-seven patients finished skin biopsy and 25 patients had positive intranuclear inclusions in skin biopsy., Positive gene and negative pathological result was found in the 2 patients. The demographic and clinical presentations were summarized in Table 1.

**Table 1:** Summary of demographic and clinical information

**Categories**	Items	Data
**Demographic information**	Sex	Male: female = 12:20
	Age onset (years, $\overline{\mathrm{x}}\pm \mathrm{s }$)	54.3 $\pm$ 10.2
	Duration of disease (months, $\overline{\mathrm{x}}\pm \mathrm{s }$)	57.8 $\pm$ 51.4
	Family history (%)	9/32 (28.1%)
**Clinical presentations**	Dementia (%)	27/32 (84.4%)
	Autonomic nervous system dysfunction (%)	22/32 (68.8%)
	*Urinary and/or fecal dysfunction (%)*	19/32 (59.4%)
	*Orthostatic hypotension (%)*	3/32 (9.4%)
	*Nausea/ vomit (%)*	3/32 (9.4%)
	*Miosis (%)*	2/32 (6.3%)
	*Hidrosis (%)*	2/32 (6.3%)
	*Erectile dysfunction (%)*	1/32 (3.1%)
	Episodes of fever (%)	12/32 (37.5%)
	Movement disorder (%)	11/32 (34.4%)
	*Parkinsonism (%)*	5/32 (15.6%)
	*Tremor (%)*	6/32 (18.8%)
	Encephalopathy like attacks (%)	11/32 (34.4%)
	Headache (%)	11/32 (34.4%)
	Stroke like attacks (%)	10/32 (31.3%)
	Seizure (%)	4/32 (12.5%)
**Examinations**	MMSE (0–30, $\overline{\mathrm{x}}\pm \mathrm{s }$)	19.6 $\pm$ 9.2
	MoCA (0–30, $\overline{\mathrm{x}}\pm \mathrm{s }$)	16.7 $\pm$ 8.9
	Abnormal electromyogram (%)	5/12 (41.7%)
	Abnormal electroencephalogram (%)	12/16 (75.0%)
	Abnormal CSF analysis (%)	0/20 (0%)
	CSF Aβ_42_ (pg/ml, $\overline{\mathrm{x}}\pm \mathrm{s }$)	882.6 $\pm$ 316.3
	CSF t-tau (pg/ml, $\overline{\mathrm{x}}\pm \mathrm{s }$)	172.4 $\pm$ 97.4
	CSF p-tau (pg/ml, $\overline{\mathrm{x}}\pm \mathrm{s }$)	52.2 $\pm$ 22.8
	Abnormal ^18^F-FDG-PET/CT (%)	2/2 (100%)

*Abbreviations*: *MMSE* Mini Mental State Examination, *MoCA* Montreal Cognitive Assessment, *CSF* Cerebrospinal fluid

Dementia, autonomic nervous system dysfunction, episodes of fever, movement disorder, encephalopathy like attacks, headache, stroke like attacks and seizure were the clinical presentations based on frequency. Encephalopathy like attacks presented as acute or subacute encephalopathy, with/without fever, consciousness disorder, confusion, delirium and hallucination, which resolved in days or weeks. Stroke like attacks presented as acute focal neurological deficit, including paraplegia, monoplegia, dizziness, numbness, paresthesia, ataxia, diplopia and dysarthria, which resolved in seconds or hours.

Cognitive impairment was around mild to moderate dementia. Abnormal electromyogram usually presented as declined motor conduction velocity and sensory conduction velocity, indicating demyelinating pathogenesis. Abnormal electroencephalogram presented as increase of slow waves, mainly in frontal and temporal leads, with/without sporadic sharp waves. All CSF analysis, including cell count, protein, glucose and cytology were normal. CSF Aβ_42_, t-tau, p-tau were examined in 6 patients, mostly in normal range, while t-tau was elevated in 1 patient. FDG-PET performed in 2 patients showed low metabolism in global cortex, significantly in temporal and parietal lobe as well as cingulate gyrus.

### Neuroimaging results

**Fig. 1:**
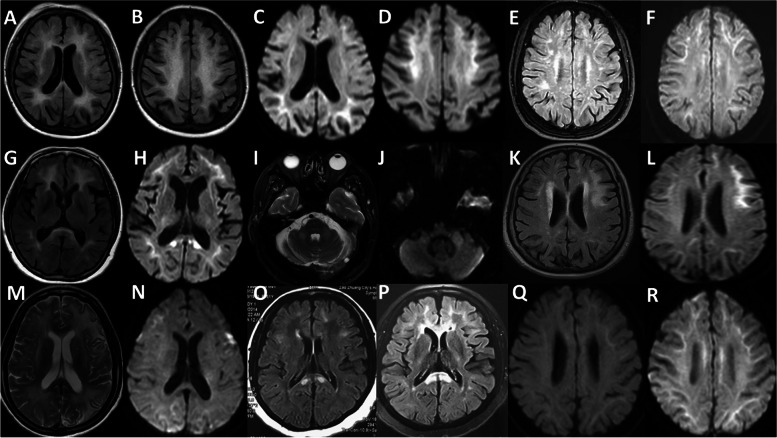
**A**-**D** F/64, widespread leukoencephalopathy with frontal lobe predominance and corticomedullary junction DWI high intensity, involving corpus callosum and external capsule. **E**–**F** F/55, patchy frontal lobe leukoencephalopathy with corticomedullary junction DWI high intensity. **G**-**H** F/59, frontal lobe and external capsule DWI high intensity with splenium of corpus callosum lesions. **I**-**J** M/61, symmetrical lesions on brachium pontis and DWI high intensity. **K**-**L** F/54, asymmetrical distribution of frontal lobe leukoencephalopathy. **M**–**N** F/45, widespread leukoencephalopathy with grey matter involvement, left predominance. **O**-**P** F/54, progression of leukoencephalopathy after 4 years follow-up. **Q**-**R** F/51, extensive increase of corticomedullary junction DWI high signal after 2 months follow up, while no change in FLAIR sequence. (FLAIR: **A**, **B**, **E**, **G**, **K**, **O**, **P**; T2-weighted: **I**, **M**; DWI: **C**, **D**, **F**, **H**, **J**, **L**, **N**, **Q**, **R**)

Thirty-one patients finished brain MRI imaging, while 2 of them only finished DWI. 1 patient had MRI contradiction and finished brain CT, which was not included in the analysis. The features of white matter lesions were listed in Table 2. All patients had leukoencephalopathy, most of which were symmetrical. Ventricle enlargement was more severe than cortical atrophy, suggesting white matter significantly damaged. Periventricular white matter, frontal subcortical and deep white matter and corpus callosum were mostly affected (Fig. 1A-H). External capsule lesions were more common than internal capsule (Fig. 1G-H). Infratentorial white matter, especially brachium pontis and pons were affected (Fig. 1I-J). Corticomedullary junction DWI high intensity was an important feature, earliest found in frontal lobe. Frontal lobe and external capsule DWI high intensity connected to form a “kite-like” specific image (Fig. 1C, H). However, asymmetrical white matter involvement and grey matter involvement were also present (Fig. 1K-N).

**Table 2:** Summary of neuroimaging features

**Categories**	Items	Data
**Features of White matter lesions**	Periventricular white matter lesions (%)	29/29 (100%)
	Subcortical and deep white matter lesions- frontal (%)	28/29 (96.6%)
	Subcortical and deep white matter lesions- parietal (%)	21/29 (72.4%)
	Subcortical and deep white matter lesions- occipital (%)	19/29 (65.5%)
	Subcortical and deep white matter lesions- temporal (%)	13/29 (44.8%)
	Internal capsule (%)	11/29 (37.9%)
	External capsule (%)	21/29 (72.4%)
	Corpus callosum (%)	27/29 (93.1%)
	Infratentorial white matter (%)	13/29 (44.8%)
	Corticomedullary junction DWI high intensity (%)	27/31^a^ (87.1%)
	Frontal and external capsule DWI high intensity (%)	11/31 (35.5%)
	Grey matter lesion (%)	5/29 (17.2%)
	Gd Enhancement (%)	0/10 (0%)
**Visual rating scale**	GCA (0–3, median)	1
	O’Donavan scale (0–3, median)	2
	Periventricular white matter (0–6, $\overline{\mathrm{x}}\pm \mathrm{s }$)	5.1 $\pm$ 1.8
	Deep white matter (0–24, $\overline{\mathrm{x}}\pm \mathrm{s }$)	16.1 $\pm$ 7.2
	Basal ganglia white matter (0–30, $\overline{\mathrm{x}}\pm \mathrm{s }$)	1.5 $\pm$ 2.2
	Infratentorial white matter (0–24, $\overline{\mathrm{x}}\pm \mathrm{s }$)	1.8 $\pm$ 2.2
	White matter total score (0–84, $\overline{\mathrm{x}}\pm \mathrm{s }$)	24.4 $\pm$ 11.3

*Abbreviations*: *DWI* Diffusion weighted imaging, *GCA* General cortical atrophy

^a^29 patients had intact MRI sequences and 2 patients only had DWI

Dynamic changes were found in 5 patients, while it remained stable for many years in 3 patients. Progressive spread of lesions and DWI high intensity were both present (Fig. 1O-R).

### Relationship between clinical presentations and neuroimaging

**Fig. 2:**
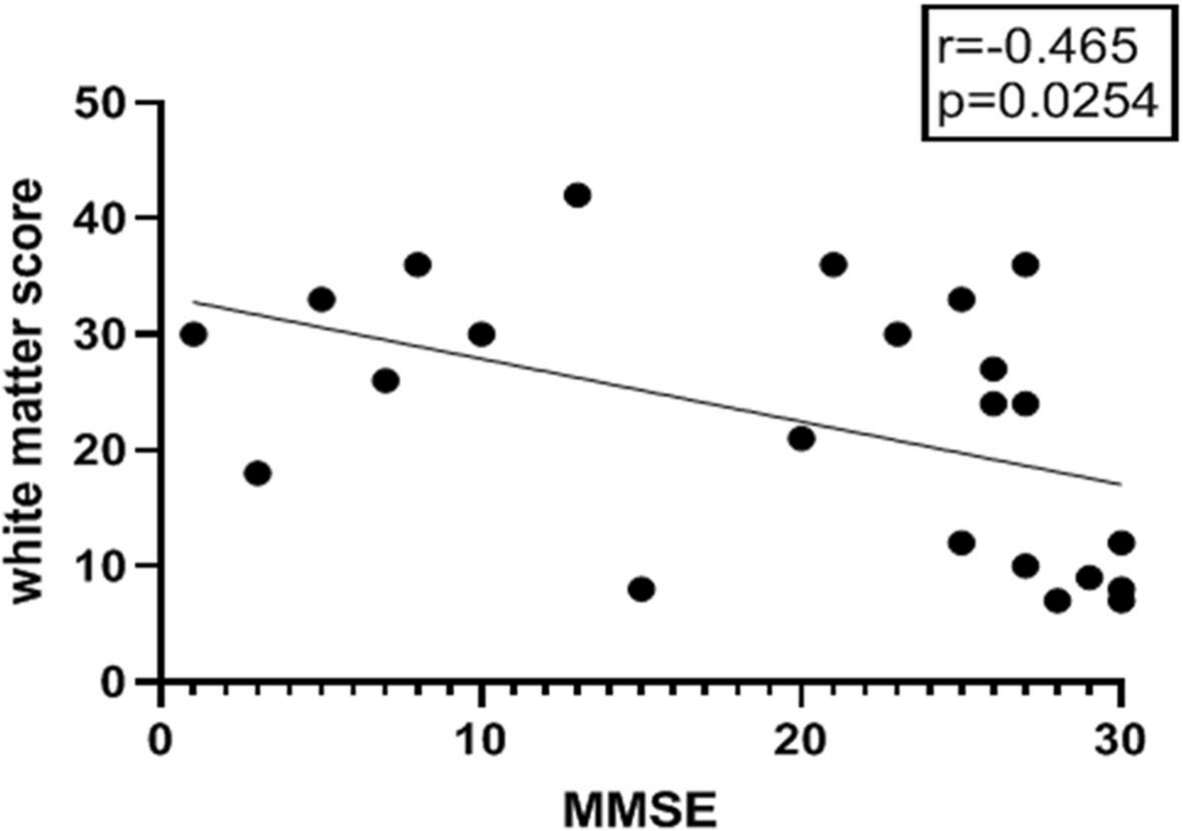
severity of white matter lesions (higher total score) was associated with severity of dementia (lower MMSE score)

Spearman’s correlation was conducted to analysis the relationship between white matter total score and duration of disease, white matter total score and severity of dementia revealed by MMSE score. White matter total score was not correlated with duration of the disease (*p* = 0.875). White matter total score was correlated with severity of dementia (*p* = 0.0067), and linear correlation was found (*r* = 0.465, *p* = 0.0254, Fig. 2).

Kruskal Wallis analysis was conducted to evaluate severity of dementia between different groups of cortical atrophy (GCA score) and ventricular enlargement (O’Donavan score). Severity of dementia was not consistent with cortical atrophy (*p* = 0.555) and ventricular enlargement (*p* = 0.583).

Mann–Whitney test was conducted to compare the basal ganglia white matter score and infratentorial white matter score with movement symptoms. Movement symptoms were not associated with basal ganglia (*p* = 0.179) and infratentorial (*p* = 0.394) white matter lesions.

χ2 test was conducted to analysis relationship between grey matter involvement and episodic attack symptoms. Stroke like attack was not related to grey matter lesion (*p* = 0.356). Encephalopathy like attack was significantly associated with grey matter involvement (*p* = 0.00077).

### Clinical-neuroimaging-pathological relationship analysis

**Fig. 3:**
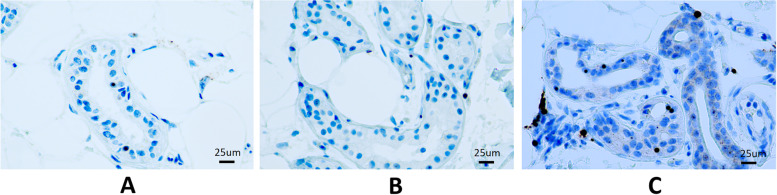
skin biopsy, anti-p62 stain, 400 × . **A** < 5 inclusion bodies. **B** 5–10 inclusion bodies. **A** > 10 inclusion bodies

We calculated the largest number of anti-p62 positive inclusion bodies in a 400 × magnified visual field, and graded into 3 subgroups: < 5, 5–10, > 10 (illustrated in Fig. 3). Then we analysis the relationship between number of inclusion bodies with duration of the disease, severity of leukoencephalopathy and severity of dementia using Kruskal Wallis analysis. We found duration of the disease (*p* = 0.561), white matter total score (*p* = 0.283) and MMSE score (*p* = 0.687) were all not associated with the amount of inclusion bodies.

## Discussion

NIID is a heterogeneous disease spectrum with systemic eosinophilic hyaline intranuclear inclusions in nervous system and visceral organ cells [13]. Skin pathology and GGC repeat evaluation are important diagnostic method of NIID. However, the mechanism and content of the inclusion bodies are not well known, and is found relating to ubiquitin-mediated protein degradation [13]. Zhong et al. found that *NOTCH2NLC* 5’UTR triggered the translation of a polyglycine (polyG)-containing protein, N2NLCpolyG, which accumulated in p62-positive inclusions in cultured cells, mouse models, and NIID patient tissues, impairing nuclear lamina and nucleocytoplasmic transport [14]. Boivin et al. also suggested a polyglycine-expanded protein, was translated from an expansion of GGC repeats, defining polyG was the culprit of mechanism in NIID [15]. However, Chen et al. reported the *NOTCH2NLC* repeat expansion was a rare cause of NIID in Europeans and that at least two distinct disease entities existed under the name NIID, suggesting NIID was genetically heterogeneous [16]. Besides, a series of neurodegenerative disorders including Parkinson’s disease, Alzheimer’s disease, frontotemporal dementia, amyotrophic lateral sclerosis et al. were found to associate with GGC repeat expansion in *NOTCH2NLC* [17]. In clinical settings, there was also a report that skin biopsy and GGC expansion testing in *NOTCH2NLC* were both negative in autopsy confirmed infantile NIID patient [18]. So extensive differential diagnosis was necessary, especially fragile X-associated tremor-ataxia syndrome (FXTAS), which was reported clinically and pathologically mimicking NIID [19, 20].

In our cohort, clinical manifestations of NIID could be generally classified into three groups: progressive neurodegenerative symptoms, autonomic system dysfunction and episodic attacks. However, the symptoms could overlap in single patient and in one pedigree. Progressive dementia was most prevalent in our cohort, which was also reported in another cohort [21]. Memory loss and frontal cognitive dysfunction was early and prominent symptom, along with predominantly frontal involvement of white matter [22]. Severe and global cognitive dysfunction was found in later stage of the disease. Besides, tremor was an important extrapyramidal presentation in NIID, which could be early onset and mimicking essential tremor, leading to missed diagnosis [21, 23]. Autonomic nervous system involvement was characteristic in NIID as in our cohort. Urinary dysfunction including urinary retention and incontinence was most common and cases with long term history of neurogenic bladder dysfunction were diagnosed NIID after more than 10 years [24]. Orthostatic hypotension, digestive tract symptoms including constipation, vomit, diarrhea, miosis, hidrosis and erectile dysfunction were all found in NIID [21, 25]. The last group was episodic attacks, including migraine like headache, seizure, stroke like and encephalopathy like attacks. Stroke and encephalopathy like attacks were rare presentations and easily lead to misdiagnosis. However, in our cohort, they were not rare. Stroke like attack presented as acute focal neurological deficit, including paraplegia, paresthesia, ataxia, diplopia and dysarthria, which resolved in seconds or hours, as reported in literature [26]. Encephalopathy like attack presented as acute or subacute encephalopathy, with/without fever, consciousness disturbance, delirium and hallucination, cognitive decline, which resolved in days or weeks, as reported [27]. In rare conditions, the two attacks could overlap in a single patient [28].

Typical head MRI of NIID showed leukoencephalopathy in T2 and FLAIR image, usually confluent and bilaterally symmetrical, generally prominent in the frontal lobe [3]. Corpus callosum might be the earliest lesions in the disease and then spread to subcortical white matter [2]. External capsule was usually involved [3]. On DWI, high intensity signal along the corticomedullary junction was found in most NIID patients, and spread along the corticomedullary junction. Usually, the DWI high intensity signal was slightly observed in regional corticomedullary junction in the frontal lobe in early stages [3, 29]. Typical neuroimaging feature was also found in our cohort. We further emphasized that frontal predominant leukoencephalopathy with corpus callosum and external capsule involvement, as well as DWI high intensity along corticomedullary junction and external capsule (kite-like sign) was suggestive MRI feature of NIID in clinical diagnosis. Symmetrical lesions on brachium pontis with DWI high intensity were also common in NIID, but should differentiate with FXTAS [20]. The dynamic evolution of MRI presentation was also interesting. In literature, DWI high intensity could appear later than clinical presentation and leukoencephalopathy, and also, it could disappear after persistent for many years, while progressive leukoencephalopathy was observed [30, 31]. However, in our cases, persistent DWI high intensity with no progression was found. Also, progressive leukoencephalopathy without concurrent DWI high intensity progression, as well as progressive DWI high intensity without concurrent leukoencephalopathy progression were both found. This meant the relationship between leukoencephalopathy and DWI high intensity need further clarification.

The severity of leukoencephalopathy was not related to disease duration in our analysis, probably because leukoencephalopathy was not symptomatic in early stage. Actually, part of NIID patients found leukoencephalopathy by occasional head MRI examination. We found severity of dementia was significantly associated with leukoencephalopathy, but not with cortical atrophy and ventricular enlargement. It was reported that Fazekas scores were significantly correlated to the global cognition, executive and language functions in NIID [32]. We found ventricular enlargement was more severe than cortical atrophy, suggesting it was probably secondary to white matter volume loss. Our result also supported the subcortical mechanism of dementia in NIID, implying leukoencephalopathy was a key manifestation in the pathogenesis of NIID. However, intranuclear inclusion bodies were widespread in both neurons and glia in autopsy cases [33]. In this condition, we still need more pathological evidence to reveal the actual pathogenesis of this rare disease. Besides, grey matter lesions were also found in NIID. We found that grey matter lesions, usually asymmetrical involvement, were significantly associated with encephalopathy like attacks in clinical course. This clinical presentation along with the MRI features caused misdiagnosis of NIID into viral encephalitis. The mode of grey matter lesions was also reported in other case series in literature, and some of the lesions were enhanced with Gd contrast media [3, 34]. Functional image research also found that in acute phase, the lesions in cortex were hypermetabolic and with increased blood flow, which reduced in chronic phase [35].

Eosinophilic intranuclear inclusions were definitive pathological changes of NIID. The distribution of inclusions was systemic and involving different kinds of cells [36]. Skin biopsy revealing intranuclear inclusions in sweat gland cells, adipocytes, fibroblasts significantly helped early and precise diagnosis of NIID [3, 37, 38]. However, we also found intranuclear inclusions in smooth muscle cells in vasculature and arrector pili muscle. Pathological studies also found areas of focal spongiosis in the subcortical white matter proximal to U-fibers, which corresponded to DWI high intensity on MRI. Besides, patchy myelin pallor and decreased density of astrocytes were found in affected white matter [33]. In the autopsy case, there was no difference in the density of p62 immunoreactive intranuclear inclusions between the impaired and preserved white matter areas [33]. In our cohort, we also found that the density of intranuclear inclusions in skin biopsy was not associated with disease duration, severity of leukoencephalopathy and dementia. In this condition, we hypothesized that intranuclear inclusions’ distribution in skin and brain were not parallel. Therefore, relationship between intranuclear inclusions in postmortem brain tissue and clinical presentations need to be examined.

It was not without limitations in our study. Firstly, the patients were recruited from dementia and leukoencephalopathy clinic, so there were no patients with onset symptoms involving peripheral neuropathy and myopathy. Secondly, sporadic cases were more common in our cohort and no pedigree information was collected, which interrupt the systemic research of familial NIID. Thirdly, as a tertiary general hospital in Beijing, follow-up was not easy for patients from all over the country. Further longitudinal follow up of the patients might be helpful to reveal the complicated clinical map of NIID.

## Conclusions

In conclusion, firstly, we described a series of NIID patients diagnosed by skin pathology and/or genetic analysis. Dementia, autonomic nervous system dysfunction and episodic attacks were three main manifestations. CSF analysis including Aβ_42_ and tau level was normal in NIID. Secondly, frontal predominant leukoencephalopathy with corpus callosum and external capsule involvement, as well as DWI high intensity along corticomedullary junction and external capsule (kite-like sign) was suggestive feature of NIID in clinical diagnosis. We found ventricular enlargement was more severe than cortical atrophy, suggesting it was probably secondary to whiter matter volume loss, and severity of dementia was correlated with leukoencephalopathy, supporting the subcortical mechanism of dementia in NIID. We also found grey matter lesions were significantly associated with encephalopathy like attacks in clinical course. Thirdly, the density of intranuclear inclusions in skin biopsy was not associated with disease duration, severity of leukoencephalopathy and dementia. Probably intranuclear inclusions’ distribution in skin and brain were not parallel in individual patients.

## Data Availability

The datasets used and/or analysed during the current study are available from the corresponding author on reasonable request.

## Abbreviations

NIID: Neuronal intranuclear inclusion disease
MRI: Magnetic resonance imaging
DWI: Difusion-weighted imaging
FLAIR: Fluid attenuated inversion recovery
MMSE: Mini–Mental State Examination
MoCA: Montreal Cognitive Assessment
ADL: Activities of daily living
FDG-PET: Fluorodeoxyglucose-positronemission computed tomography
GCA: General cortical atrophy
EDTA: Ethylene diamine tetra acetic
FXTAS: Fragile X-associated tremor-ataxia syndrome
CSF: Cerebrospinal fuid
